# A statistical method for the conservative adjustment of false discovery rate (*q*-value)

**DOI:** 10.1186/s12859-017-1474-6

**Published:** 2017-03-14

**Authors:** Yinglei Lai

**Affiliations:** Department of Statistics and Biostatistics Center, The George Washington University, Washington D.C., 20052 USA

**Keywords:** False discovery rate, *q*-value, Conservative adjustment

## Abstract

**Background:**

*q*-value is a widely used statistical method for estimating false discovery rate (FDR), which is a conventional significance measure in the analysis of genome-wide expression data. *q*-value is a random variable and it may underestimate FDR in practice. An underestimated FDR can lead to unexpected false discoveries in the follow-up validation experiments. This issue has not been well addressed in literature, especially in the situation when the permutation procedure is necessary for *p*-value calculation.

**Results:**

We proposed a statistical method for the conservative adjustment of *q*-value. In practice, it is usually necessary to calculate *p*-value by a permutation procedure. This was also considered in our adjustment method. We used simulation data as well as experimental microarray or sequencing data to illustrate the usefulness of our method.

**Conclusions:**

The conservativeness of our approach has been mathematically confirmed in this study. We have demonstrated the importance of conservative adjustment of *q*-value, particularly in the situation that the proportion of differentially expressed genes is small or the overall differential expression signal is weak.

## Background

Microarray and sequencing technologies have been widely used in genome-wide expression experimental for biological and medical studies [1–5]. After screening a large number of genes simultaneously, we expect to achieve new biological discoveries. In these situations, an important statistical concept is multiple hypothesis testing, in which many statistical tests are conducted at the same time. Then, a detection of gene with relatively small *p*-value may be actually a false discovery. Since the introduction of microarray technology, the concept of false discovery rate (FDR) and its related statistical methods have been well developed [6, 7]. *q*-value is a statistical method for the estimation of FDRs [8]. It has been widely used in the analysis of microarray and sequencing data.

Since *q*-value is an estimation method, it is possible that it underestimates true FDRs. Then, an undesirable consequence is that many genes detected with low *q*-value cannot be validated in a follow-up experiment. Therefore, it is important to avoid underestimations of FDRs. However, there is a lack of statistical method to address this important issue. Furthermore, in many situations, *q*-values are calculated based on *p*-values that are evaluated based on a permutation procedure (due to unknown data distributions or non-traditional test statistics). Then, these *p*-values are also estimated. This increases the complexity of FDR underestimations. It is necessary to adjust the underestimation of FDRs in a comprehensive approach.

In this study, we proposed a statistical method for the conservative adjustment of *q*-value, which is one of the most frequently used procedure for estimating FDRs. We first reviewed the concepts of multiple hypothesis testing, FDR and *q*-value. Then, we introduced a concept of conservative adjustment. Based on the theory of rank-based quantiles, we described a non-parametric approach and we conducted simulation and application studies to illustrate its usefulness.

## Methods

### Multiple hypothesis testing and false discovery rate

When a large number of variables are simultaneously screened, it is usually a situation that a mixture of true and false null hypotheses is presented. (There is a hypothesis to test for each variable but the underlying true/false nature is unknown). Statistically, this is a situation of multiple hypothesis testing (MHT). An illustrative summary is shown in Table 1. After certain statistical tests for *m* total hypotheses, with a threshold for declaring significance, we have *R* hypotheses rejected (claimed positives). If we know the underlying nature of each variable, then its related hypothesis can be classified as either a true null or a false null (termed gold standard). If this information is provided, then we know the numbers *U*, *V*, *W* and *S* in Table 1. *U*, *V*, *W* and *S* represent the numbers of true negatives, false positives, false negatives and true positives, respectively. However, in practice, the gold standard information (or the underlying nature) is usually unknown. Therefore, it is statistically interesting to evaluate *U*, *V*, *W* or *S* (or their combinations).

**Table 1:** A summary in the situation of multiple hypothesis testing

	True null	False null	Total
Negative	*U*	*W*	*m*−*R*
Positive	*V*	*S*	*R*
Total	*m*_0_	*m*−*m*_0_	*m*

This table shows the numbers of true/false negatives/positives in the situation of multiple hypothesis testing. The details are described in the Methods section

The traditional family-wise error rate (FWER) provides a strong control on *V* [9]. Since FWER is too conservative (for example, requiring extremely small *p*-value threshold), it is usually difficult to claim statistical significance. The false discovery rate (FDR) has been proposed to empirically evaluate the proportion of false positives among the claimed positives: *V*/*R* [6]. The concept of FDR and its related estimations have been widely used in the analysis of genome-wide expression data collected by microarray or RNA sequencing technologies. Particularly, *q*-value [8] is one of the most widely used method for FDR estimation.

### *q*-Value

Storey and Tibshirani [8] proposed the *q*-value method that is a statistical procedure for FDR evaluation. Suppose *T* is the test statistic and genes with test scores greater than or equal to *t* will be claimed significant. Let *α* be the *p*-value at *t*, and let *f*(*t*) and *s*(*t*) be the expected numbers of false positives and significant genes, respectively. Storey and Tibshirani [8] proposed that the FDR for *T*=*t* could be approximated by the proportion of false positives:

1 $$\begin{array}{@{}rcl@{}} FDR(t) &\approx& f(t)/s(t) \\  &=& m \pi_{0} \mathbf{Pr}(T \ge t | H_{0}) / [m \mathbf{Pr}(T \ge t)], \end{array} $$

where *m* is the total number of genes and *π*_0_ is the proportion of true null hypotheses (i.e. *π*_0_=*m*_0_/*m* in Table 1). With a proper estimator $\hat {\pi }_{0}$ for *π*_0_, they proposed the *q*-value as a FDR estimator:

$$q(t)=m \hat{\pi}_{0} \alpha / (\#\{T \ge t \}), $$

where (*#*{*T*≥*t*}) estimates *m* **Pr**(*T*≥*t*).

### Conservative adjustment of *q*-value

In practice, it can be difficult to obtain the theoretical *p*-value of *t*. Therefore, a permutation based *p*-value $\hat {\alpha }$ has been widely used to estimate *α*. Then, the permutation version of *q*-value is

$$q(t)=m \hat{\pi}_{0} \hat{\alpha} / (\#\{ T \ge t\}). $$

When the permutation method [10] is used to evaluate *p*-values, it is possible to obtain underestimated results. We have proposed a conservative adjustment of permutation *p*-values to address this issue [11].

Similarly, the above FDR can be underestimated since a *q*-value is actually a combination of three estimates:

- $\hat {\pi }_{0}$ for *π*_0_,
- $\hat {\alpha }$ for *α*,
- (*#*{*T*≥*t*}) for *m* **Pr**(*T*≥*t*).

From above, notice that *m* **Pr**(*T*≥*t*) must still be empirically estimated even when *α* can be theoretically determined. To address the underestimation of FDR (from *q*-value), our solution is to consider a conservative adjustment of *q*(*t*).

According to Eq. (1), the theoretical *q*-value for *T*=*t* can be defined as:

$$\tilde{q}(t) = \pi_{0} \alpha(t) / \gamma(t), $$

where *α*(*t*)=**Pr**(*T*≥*t*|*H*_0_) and *γ*(*t*)=**Pr**(*T*≥*t*).

We define the 100(1−*a*)*%* conservative adjustment of *q*(*t*) as an estimator $\hat {q}_{c}(t) \in [0,1]$ such that:

$$\mathbf{Pr}[\hat{q}_{c}(t) \ge \tilde{q}(t)] \ge 1-a. $$

Our solution for $\hat {q}_{c}(t)$ is to find $\hat {\pi }_{0c}$, $\hat {\alpha }_{c}(t)$ and $\hat {\gamma }_{c}(t)$ such that:

$$\begin{array}{@{}rcl@{}} \mathbf{Pr}(\hat{\pi}_{0c} \ge \pi_{0}) &\ge& 1-a_{0}; \\ \mathbf{Pr}[\hat{\alpha}_{c}(t) \ge \alpha(t)] &\ge& 1-a_{1}; \\ \mathbf{Pr}[\hat{\gamma}_{c}(t) \le \gamma(t)] &\ge& 1-a_{2}. \end{array} $$

If (1−*a*_0_)(1−*a*_1_)(1−*a*_2_)≥1−*a*, then we claim that $\hat {\pi }_{0c} \hat {\alpha }_{c}(t) / \hat {\gamma }_{c}(t)$ satisfies the requirement for $\hat {q}_{c}(t)$, given *π*_0_,*α*(*t*)>0 and $\mathbf {Pr}[\hat {\gamma }_{c}(t)>0]=1$. The mathematical proof is given as an Appendix 1.

#### **Remark**

$\hat {q}_{c}(t)$ is actually an upper confidence limit of $\tilde {q}(t)$. However, due to the difficulty in the accurate estimation of *π*_0_ (*π*_0_ is usually conservatively estimated), it is difficult to calculate a lower confidence limit of $\tilde {q}(t)$ in practice.

### Conservative estimation of *π*_0_

Due to the identifiability issue, *π*_0_ (the proportion of true null hypotheses) is usually conservatively estimated in practice [12]. Many statistical methods have been proposed for the estimation of *π*_0_ [13]. Among them, the **convest** [14] is a well-recognized method that conservatively estimates *π*_0_. (Instead of estimating the true *π*_0_, it estimates a close upper bound of *π*_0_. Therefore, the estimation is conservative since the proportion of non-differentially expressed genes is usually overestimated in a differential expression analysis). According to our experience, it is reasonable to assume that

$$\mathbf{Pr}(\hat{\pi}_{0c} \ge \pi_{0}) \approx 1, $$

When $\hat {\pi }_{0c}$ is the **convest** method (or other similar methods). Then, we discuss $\hat {\alpha }_{c}(t)$ and $\hat {\gamma }_{c}(t)$, which are closely related to some rank-based quantiles.

### Conservative adjustment of rank-based quantiles

In a differential expression analysis, only *q*-values associated with observed test scores can be evaluated, and it is difficult to accurately evaluate the *q*-values for unobserved test scores. Here are the mathematical details. With a test statistic *T*, we can obtain *m* observed test scores {*T*_1_,*T*_2_,…,*T*_*m*_} for *m* variables (genes). An empirical estimate of *γ*(*t*)=**Pr**(*T*≥*t*) is

$$\hat{\gamma}(t)=\sum_{i=1}^{m} \delta(T_{i} \ge t) / m, $$

where the indicator function *δ*(TRUE)=1 and *δ*(FALSE)=0. If we sort these *m* test scores in an increasing order: *T*_(1)_≤*T*_(2)_≤…≤*T*_(*m*)_, then we have

$$\hat{\gamma}(T_{(i)})= (m-i+1)/m, $$

which is a rank-based quantile estimator.

If the theoretical distribution of *T* is unknown, then we need to use the permutation procedure [10] for evaluating *p*-values. In the permutation procedure, we permute sample labels and recalculate test scores. Since the observed test scores (scores calculated based the original data) can also be considered as the results from a particular permutation, they are generally included in the pool of permuted test scores. Based on *r* permuted test scores: $\{ T^{0}_{1}, T^{0}_{2}, \ldots, T^{0}_{r} \}$, an empirical estimate of *α*(*t*)=**Pr**(*T*≥*t*|*H*_0_) is

$$\hat{\alpha}(t)=\sum_{i=1}^{r} \delta(T^{0}_{i} \ge t) / r. $$

If we sort these *r* permuted test scores in an increasing order: $T^{0}_{(1)} \le T^{0}_{(2)} \le \ldots \le T^{0}_{(r)}$, then we have

$$\hat{\alpha}(T^{0}_{(i)})=(r-i+1)/r. $$

Note that, if the observed test scores are not included in the pool of permuted test scores, then the permutation *p*-value of *T*=*t* will be $\hat {\alpha }(T^{0}_{(k)})=(r-k+1)/r$, where $T^{0}_{(k)} \ge t$ is the closest order statistic to *t*. Therefore, the permutation *p*-value of *T*=*t* can also be considered as a rank-based quantile estimator.

Since $\hat {\gamma }$ and $\hat {\alpha }$ are both random variables, it is possible for them to underestimate their target parameters. The above discussion shows that, in practice, $\hat {\gamma }$ and $\hat {\alpha }$ are actually both rank-based quantile estimators. Based on the theory of order statistics [15], we have proposed a conservative adjustment for this type of estimator [11]. Such an adjustment requires no parametric assumption on the distribution of test statistic and the solution can be expressed by a normalised incomplete beta function. Therefore, based on this adjustment, we can otain $\hat {\alpha }_{c}(t)$ and $\hat {\gamma }_{c}(t)$ such that $\mathbf{Pr}[\hat {\alpha }_{c}(t) \ge \alpha (t)] \ge 1-a_{1}$ and $\mathbf{Pr}[\hat {\gamma }_{c}(t) \le \gamma (t)] \ge 1-a_{2}$.

## Results

### A simulation study

To understand how likely the *q*-value method underestimates the true false discovery rate, we conducted a simulation study. We choose the normal distributions for simulating expression data and the Student’s *t* for differential expression. In this way, we could evaluate the true false discovery rate theoretically.

Ten thousand genes were simulated for two sample groups with sample size 5 for each group (10 for total sample size). For non-differentially expressed genes, the expression data were simulated from *N*(0,1) for both groups. Then, we considered four scenarios. For the first simulation scenario, the proportion of differentially expressed genes was 10%; the expression data for differentially expressed genes were simulated from *N*(0,1) and *N*(1,1) for the first and the second sample groups, respectively (*Δ*=1). For the second simulation scenario, the proportion of differentially expressed genes was 10%; the expression data for differentially expressed genes were simulated from *N*(0,1) and *N*(2,1) for the first and the second sample groups, respectively (*Δ*=2). For the third simulation scenario, the proportion of differentially expressed genes was 20%; the expression data for differentially expressed genes were simulated from *N*(0,1) and *N*(1,1) for the first and the second sample groups, respectively (*Δ*=1). For the last simulation scenario, the proportion of differentially expressed genes was 20%; the expression data for differentially expressed genes were simulated from *N*(0,1) and *N*(2,1) for the first and the second sample groups, respectively (*Δ*=2).

For all different scenarios, the Student’s *t*-test was used for differential expression analysis. To evaluate *p*-values, we performed all possible (126) permutations for each simulated gene and pooled all 1,260,000 permuted test scores together as the empirical null distribution. (In practice, the underlying data distributions are unknown and the permutation procedure is widely used.) For each scenario, we conducted 100 repetitions to understand the variations in the simulation results.

**Fig. 1:**
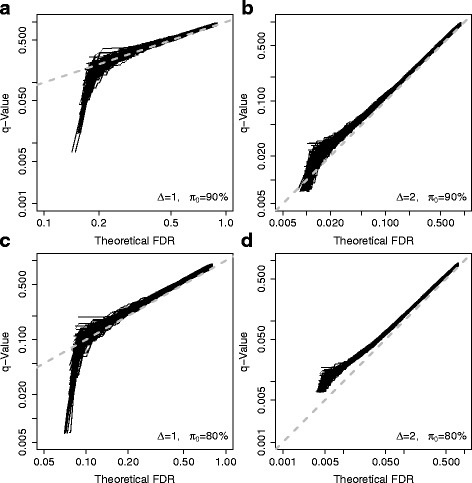
Simulation results for four scenarios. **a** Relatively weak differential expression and relatively small proportion of differential expression. **b** Relatively strong differential expression but relatively small proportion of differential expression. **c** Relatively weak differential expression but relatively large proportion of differential expression. **d** Relatively strong differential expression and relatively large proportion of differential expression. The simulation details are described in the Results section

**Fig. 2:**
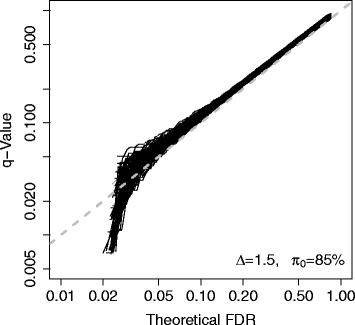
Simulation results for a typical scenario. Moderate differential expression and moderate proportion of differential expression. The simulation details are described in the Results section

The simulation results are shown in Fig. 1. For each scenario, the theoretical true FDRs is compared to their related estimates (*q*-values). In summary, when the proportion of differentially expressed genes (1−*π*_0_) becomes smaller (from 20 to 10%), it is more likely to obtain underestimated FDRs; when the signal of differential expression (*Δ*) becomes weaker, it is more likely to obtain underestimated FDRs. Figure 2 gives a scenario with a moderate proportion of differentially expressed genes (1−*π*_0_=15*%*) and overall moderate differential expression signals (*Δ*=1.5). Each curve is a comparison between the empirical FDRs (*q*-values) vs. theoretical FDRs (based on one repetition of simulation). Below 0.05 theoretical FDR, some curves can be observed under the diagonal line, which indicate that these empirical FDRs (*q*-values) are underestimated. Furthermore, among 100 repetitions, there is a considerable portion of empirical FDRs underestimated (when the theoretical FDR below 0.05).

### An artificial illustration

A conservative adjustment of false discovery rate (FDR) can be useful in practice, especially before the experimental validation of genes identified from a genome-wide expression study. For example, based on a microarray or RNA-seq study, one may want to validate a few genes with *q*-value less than 10%. However, it may be surprising that much less genes can be confirmed after the RT-PCR validation. (The validation result is beyond our expectation based on 5% estimated FDR.) This hypothetical situation would be an example of under-estimation of FDR.

**Fig. 3:**
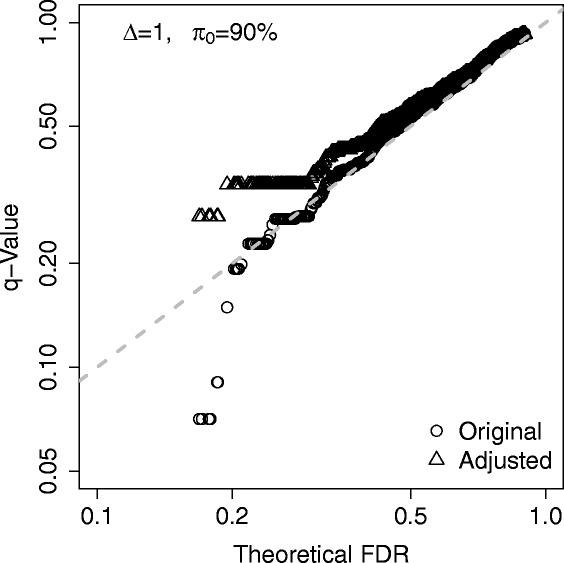
A simulation example for an artificial illustration. The theoretical true false discovery rate (FDR) is compared to the related estimate by *q*-value. This is a scenario with relatively weak differential expression and relatively small proportion of differential expression. Dark circles represent original (unadjusted) *q*-values and dark triangles represent conservatively adjusted *q*-values. The simulation details are described in the Results section

To demonstrate the above situation, we conducted a simple simulation study. 10,000 genes were simulated for two sample groups. The sample size was 5 for each group. The proportion of differentially expressed genes was 10%. The expression profiles of differentially expression genes were simulated from *N*(0,1) and *N*(1,1) for the first and the second sample groups, respectively. The expression profiles of non-differentially expressed genes were simulated from *N*(0,1) for both groups. The Student’s *t*-test was used for differential expression analysis. To evaluate *p*-values, we performed all possible (126) permutations for each simulated gene and pooled all 1,260,000 permuted test scores together as the empirical null distribution. (In practice, the underlying data distributions are unknown and the permutation procedure is widely used.) The **convest** method proposed by Langaas et al. [14] was used to obtain an estimated *π*_0_, which was used in the *q*-value estimation procedure [8]. In this simulation it is feasible to calculate the true FDR theoretically. Figure 3 shows that the low values of true FDR can be seriously under-evaluated by *q*-value. Then, we considered a conservative adjustment. We set *a*_0_=0 (since *π*_0_ is usually conservatively estimated), $a_{1}=1-\sqrt {0.95}$ and $a_{2}=1-\sqrt {0.95}$ (then *a*=0.05). Figure 3 shows the conservatively adjusted *q*-values.

### Three applications

We applied our method to two genome-wide expression data sets. The first one was a microarray data set and it was collected for a diabetes study. It is well-known that differential expression signals are usually weak in diabetes studies. When the sample size is not relatively large, it is usually difficult to detect true differentially expressed genes. (Due to the inflated false positive rates from the multiple hypothesis testing for a large number of genes, genes with seemingly small FDRs are likely noise genes). It is interesting to understand the adjustment effects from our method for this scenario. The second one was a RNA sequencing (RNA-seq) data set and it was collected for a prostate cancer study. It is also well-known that differential expression signals are usually strong in cancer studies. Genome-wide expression data for different types of cancer have been increasingly collected in The Cancer Genome Atlas project [3]. The current sample sizes in many TCGA cancer studies are relatively large. Then, it is also interesting to understand the adjustment effects from our method for such as scenario.

In practice, it is not feasible to calculate the theoretical true FDRs. The curve of estimated FDR vs. number of identified genes is widely used to for a summary of differential expression analysis. (In this curve, the *y*-value is a specific FDR and the *x*-value is the related number of genes with the specific FDR). Our application results can also be summarized in term of this type of curve.

**Fig. 4:**
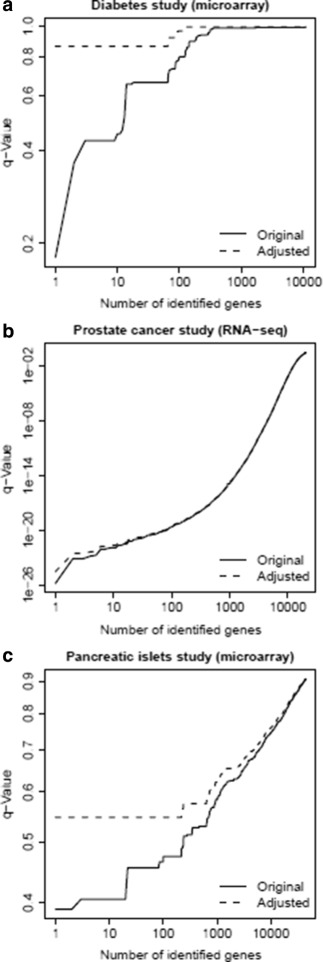
Three applications to experimental genome-wide expression data. **a** A microarray data set collected for a type 2 diabetes study. **b** A RNA sequencing (RNA-seq) data set collected for a prostate cancer study in The Cancer Genome Atlas (TCGA) project. **c** A microarray data set collected for a pancreatic islet study. The curves represent *q*-value (as estimated FDR) vs. its related number of identified genes. In each application, *dark solid curve* represents original (unadjusted) *q*-values and *dark dashed curve* represents conservatively adjusted *q*-values

For the first microarray genome-wide expression data set for a type 2 diabetes study [16], there were 17 normal subjects and 18 diabetic subjects. After the procedure of gene filtering [16], there are 10,983 genes. Based on 1,000 permutations and the related Student’s *t*-test calculations, there were 10,983,000 permuted test scores as our empirical null distribution for *p*-value evaluations. (Since it is difficult to enumerate all possible permutations, we performed 1,000 of them). We set *a*_0_=0 (since *π*_0_ is already conservatively estimated by the **convest** method [14]), $a_{1}=1-\sqrt {0.95}$ and $a_{2}=1-\sqrt {0.95}$ (then *a*=0.05). Figure 4 a shows that the *q*-values (estimated FDRs) can only be as low as slightly less than 0.2. There were only several genes with *q*-values around 0.2. However, after our conservative adjustment, Fig. 4 a shows that all the conservatively adjusted *q*-values are greater than 0.8. This comparison implies that most genes were very likely noise genes and the detections of differentially expressed genes by low *q*-values could be very unreliable. The only gene with *q*-value less than 0.2 is a mRNA for CD20-like precursor. However, no literature was found to support its association with diabetes diseases.

For the second RNA-seq genome-wide expression data set for a prostate cancer study [3], there were 52 normal subjects and 445 tumor subjects (at the time of data download for this analysis). There are 20,531 genes. Since the sample size was large and the RNA-seq expression profiles were count-type data, we used the non-parametric Wilcoxon rank sum test with its theoretical *p*-values calculation. Therefore, there was no need for an adjustment of *p*-values (i.e. *a*_1_=0). Then, we set *a*_0_=0 (since *π*_0_ is already conservatively estimated by the **convest** method [14]) and *a*_2_=1−0.95 (then *a*=0.05). Figure 4 b shows that the *q*-values (estimated FDRs) can be extremely low and lots of genes can be detected. After our conservative adjustment, Fig. 4 b shows that the curve of adjusted *q*-values almost overlaps with the curve of original *q*-values. This comparison implies that many genes were truly differentially expressed genes and the detection of these genes by low *q*-values could be highly confident. After checking literature for top ranked genes, many of them have been studied to be either directly or indirectly related to prostate cancer or general cancer diseases (details not shown).

For the third microarray genome-wide expression data set for a pancreatic-islet study [17], there were 7 normal patients and 5 type 2 diabetic patients. There are 44,928 genes and ESTs. Based on all possible permutations and the related Student’s *t*-test calculations. We set *a*_0_=0 (since *π*_0_ is already conservatively estimated by the **convest** method [14]), $a_{1}=1-\sqrt {0.95}$ and $a_{2}=1-\sqrt {0.95}$ (then *a*=0.05). Figure 4 c shows that the *q*-values (estimated FDRs) can only be as low as approximately 0.4 (about 20 genes). However, after our conservative adjustment, Fig. 4 c shows that all the conservatively adjusted *q*-values are greater than 0.5. This comparison implies that more top ranked genes were likely noise genes. The only two genes with *q*-value less than 0.4 are mRNAs for ARNT and APCDD1. Although ARNT has been widely studied for its association with diabetes diseases, no literature was found to support the association between APCDD1 and diabetes diseases.

## Discussion

For our method, there are three components that can be adjusted separately. The first component is on *π*_0_ estimation. Since the estimators for *π*_0_ are usually conservative (especially for the **convest** method [14]), we do not suggest any adjustment for this component according to our experience. The last component is on the number of identified genes. It can be adjusted based on the theory of order statistic. The second component is on *p*-values. When theoretical *p*-value can be obtained, it is not necessary to adjust this component. For permutation *p*-values, an adjustment can be also performed based on the theory of order statistic. Notice that the number of permutations is also important. In practice, we need to determine it before data analysis. When the sample size is relatively small, we can enumerate all possible permutations. When the sample size is relatively large, we can set the number of permutations as large as possible according to the computing power.

A clear advantage of our approach is that there are rigorous mathematical theories to support it. Furthermore, no distribution assumptions are required for our conservative adjustment. However, the adjustment may be over-conservative. If we could further improve the control of upper/lower bounds (as shown in our mathematical proof), then less conservative adjustment of *q*-value could be achieved. Furthermore, an independence assumption is required. It is well-known that genes work with each other during molecular and cellular processes. It would be an interesting future research topic to address the dependence among genes. Therefore, it will be our future research topics to investigate possible better upper/lower bounds for the conservative adjustment of *q*-value, as well as the impact of dependence on the conservative adjustment of *q*-value.

## Conclusions

In this study, we proposed a statistical method for the conservative adjustment of *q*-value, which is widely used to estimate false discovery rate (FDR) in practice. We provided a mathematical proof to confirm the conservativeness of our approach. We conducted simulation studies to understand how likely the *q*-value method would underestimate FDRs. From our simulation results, both the proportion of differentially expressed genes and the overall differential expression signal were two important factors. When both of them were relatively small/weak, it was likely to identify genes with underestimated FDRs. Our first application was based on a microarray diabetes study data set with relatively small sample size (and weak differential expression signals). Our third application was based on a microarray pancreatic islet study data set with relatively small sample size (and also weak differential expression signals). The results were consistent with the conclusion from our simulation studies. Our second application was based on a RNA-seq prostate cancer study data set with relatively large sample size (and strong differential expression signals). According to the results, the conservatively adjusted *q*-values were close to the originally unadjusted *q*-values.

## Appendix 1

**Mathematical Proof:**

$$\begin{array}{@{}rcl@{}} \lefteqn{\mathbf{Pr}\Bigg[\frac{\hat{\pi}_{0c} \hat{\alpha}_{c}(t) }{ \hat{\gamma}_{c}(t)} \ge \frac{\pi_{0} \alpha(t) }{ \gamma(t)} \Bigg]} \\[-1pt] &=& \mathbf{Pr}\bigg[ \frac{\hat{\pi}_{0c} \hat{\alpha}_{c}(t) }{ \hat{\gamma}_{c}(t)} \ge \frac{\pi_{0} \alpha(t) }{ \gamma(t)} \bigg| \hat{\pi}_{0c} \ge \pi_{0} \bigg]\mathbf{Pr}(\hat{\pi}_{0c} \ge \pi_{0}) \\[-1pt] && + \mathbf{Pr}\bigg[ \frac{\hat{\pi}_{0c} \hat{\alpha}_{c}(t) }{ \hat{\gamma}_{c}(t)} \ge \frac{\pi_{0} \alpha(t) }{ \gamma(t)} \bigg| \hat{\pi}_{0c} < \pi_{0} \bigg]\mathbf{Pr}(\hat{\pi}_{0c} < \pi_{0}) \\[-1pt] &\ge& \mathbf{Pr}\bigg[ \frac{\hat{\pi}_{0c} \hat{\alpha}_{c}(t) }{ \hat{\gamma}_{c}(t)} \ge \frac{\pi_{0} \alpha(t) }{ \gamma(t)} \bigg| \hat{\pi}_{0c} \ge \pi_{0} \bigg]\mathbf{Pr}(\hat{\pi}_{0c} \ge \pi_{0}) \\[-1pt] &\ge& \mathbf{Pr}\bigg[ \frac{\hat{\pi}_{0c}}{\pi_{0}} \frac{\hat{\alpha}_{c}(t) }{ \hat{\gamma}_{c}(t)} \ge \frac{\alpha(t) }{ \gamma(t)} \bigg| \hat{\pi}_{0c} \ge \pi_{0} \bigg](1-a_{0}) \\[-1pt] &=& \mathbf{Pr}\bigg[ \frac{\hat{\pi}_{0c}}{\pi_{0}} \frac{\hat{\alpha}_{c}(t) }{ \hat{\gamma}_{c}(t)} \ge \frac{\alpha(t) }{ \gamma(t)} \bigg| \frac{\hat{\pi}_{0c}}{\pi_{0}} \ge 1 \bigg](1-a_{0}) \\[-1pt] &\ge& (1-a_{0}) \mathbf{Pr}\bigg[ \frac{\hat{\alpha}_{c}(t) }{ \hat{\gamma}_{c}(t)} \ge \frac{\alpha(t) }{ \gamma(t)} \bigg]~~~~~~~~~\mathbf{Remark}\\[-1pt] &=& (1\,-\,a_{0})\! \Bigg\{ \mathbf{Pr}\bigg[ \frac{\hat{\alpha}_{c}(t) }{ \hat{\gamma}_{c}(t)} \!\ge\! \frac{\alpha(t) }{ \gamma(t)} \!\bigg| \hat{\alpha}_{c}(t) \!\ge\! \alpha(t) \bigg] \mathbf{Pr}[\hat{\alpha}_{c}(t) \!\ge\! \alpha(t)] \\[-1pt] && + \mathbf{Pr}\bigg[ \frac{\hat{\alpha}_{c}(t) }{ \hat{\gamma}_{c}(t)} \ge \frac{\alpha(t) }{ \gamma(t)} \bigg| \hat{\alpha}_{c}(t) < \alpha(t) \bigg] \mathbf{Pr}[\hat{\alpha}_{c}(t) < \alpha(t)] \Bigg\}\\[-1pt] &\ge& (1-a_{0}) \mathbf{Pr}\bigg[ \frac{\hat{\alpha}_{c}(t) }{ \hat{\gamma}_{c}(t)} \!\ge\! \frac{\alpha(t) }{ \gamma(t)} \bigg| \hat{\alpha}_{c}(t) \!\ge\! \alpha(t) \bigg] \mathbf{Pr}[\hat{\alpha}_{c}(t) \!\ge\! \alpha(t)] \\[-1pt] &=& (1-a_{0})(1-a_{1}) \mathbf{Pr}\bigg[ \frac{\hat{\alpha}_{c}(t)}{\alpha(t)} \frac{1 }{ \hat{\gamma}_{c}(t)} \ge \frac{1 }{ \gamma(t)} \bigg| \frac{\hat{\alpha}_{c}(t)}{\alpha(t)} \ge 1 \bigg] \\[-1pt] &\ge& (1-a_{0})(1-a_{1}) \mathbf{Pr}\bigg[ \frac{1 }{ \hat{\gamma}_{c}(t)} \ge \frac{1 }{ \gamma(t)} \bigg]~~~~~~~~~\mathbf{Remark}\\[-1pt] &=& (1-a_{0})(1-a_{1}) \mathbf{Pr}[ \hat{\gamma}_{c}(t) \le \gamma(t) ]\\ &=& (1-a_{0})(1-a_{1})(1-a_{2}) \end{array} $$

### **Remark**

For any random variable *X*, *Y* and a constant *c*, **Pr**(*X* *Y*≥*c*|*X*≥1)≥**Pr**(*Y*≥*c*) since *Y*≥*c*⇒*X* *Y*≥*c* given *X*≥1 ({*Y*≥*c*} is a subset of {*X* *Y*≥*c*} when *X*≥1).

## Appendix 2

R-functions for calculating conservatively adjusted *q*-values.



Notice that pi0est is a conservative estimate of *π*_0_; pvals is a list of permutation *p*-values based on *r* permutations; m is the number of permutation *p*-values (also the number of genes).
